# Challenges to implementation of the WHO Global Code of Practice on International Recruitment of Health Personnel: the case of Sudan

**DOI:** 10.1186/s12960-016-0117-8

**Published:** 2016-06-30

**Authors:** Ayat Abuagla, Elsheikh Badr

**Affiliations:** National Human Resources for Health Observatory, Federal Ministry of Health, Baladiya Street, PO Box 978, Khartoum, Sudan; Sudan Medical Specialization Board, Khartoum, Sudan

**Keywords:** Health workforce, Migration, Sudan, WHO Code

## Abstract

**Background:**

The WHO Global Code of Practice on the International Recruitment of Health Personnel (hereafter the WHO Code) was adopted by the World Health Assembly in 2010 as a voluntary instrument to address challenges of health worker migration worldwide. To ascertain its relevance and effectiveness, the implementation of the WHO Code needs to be assessed based on country experience; hence, this case study on Sudan.

**Methods:**

This qualitative study depended mainly on documentary sources in addition to key informant interviews. Experiences of the authors has informed the analysis.

**Results:**

Migration of Sudanese health workers represents a major health system challenge. Over half of Sudanese physicians practice abroad and new trends are showing involvement of other professions and increased feminization. Traditional destinations include Gulf States, especially Saudi Arabia and Libya, as well as the United Kingdom and the Republic of Ireland. Low salaries, poor work environment, and a lack of adequate professional development are the leading push factors. Massive emigration of skilled health workers has jeopardized coverage and quality of healthcare and health professional education. Poor evidence, lack of a national policy, and active recruitment in addition to labour market problems were barriers for effective migration management in Sudan. Response of destination countries in relation to cooperative arrangements with Sudan as a source country has always been suboptimal, demonstrating less attention to solidarity and ethical dimensions.

The WHO Code boosted Sudan’s efforts to address health worker migration and health workforce development in general. Improving migration evidence, fostering a national dialogue, and promoting bilateral agreements in addition to catalysing health worker retention strategies are some of the benefits accrued. There are, however, limitations in publicity of the WHO Code and its incorporation into national laws and regulatory frameworks for ethical recruitment. The outlook is bleak for Sudan unless the country designs and implements a robust national policy for migration management and unless prospects for source–destination country collaboration improve within a more sound version of the WHO Code.

**Conclusions:**

The WHO Code catalysed some vital steps in managing migration and strengthening the national health workforce in Sudan. Nevertheless, the country has not utilized the full potential of this instrument. Revisions of the WHO Code would benefit much from lessons of its application in the context of developing countries such as Sudan.

## Background

The WHO Global Code of Practice on the International Recruitment of Health Personnel (hereafter the WHO Code) was adopted by the World Health Assembly in 2010 as a voluntary instrument to assist in addressing the challenges of health worker migration and health workforce development worldwide [1]. The WHO Code urges both source and destination countries to promote ethical principles and measures to maximize gains and mitigate adverse effects of the international migration of health workers. Specifically, the WHO Code suggests several actions, including exchange of information, cooperative arrangements, and joint efforts by source and destination countries to ensure health workforce sustainability and observe the rights and interests of individual health workers. The WHO Code stipulated a number of implementation and monitoring arrangements with the aim of addressing the challenges posed by health worker mobility on health systems, especially in developing countries.

Nearly 5 years after its adoption, there is a need to review the WHO Code and its implementation by countries to ascertain the barriers to and derive lessons for the international mobility of health workers. This ought to be a priority review area, especially since some voices are increasingly questioning the relevance and effectiveness of the WHO Code [2, 3]. Additionally, the WHO Code document itself affirms the dynamicity of its content and the need for further review and amendment based on assessments of its implementation [1].

The case study presented herein aims to reflect on the dynamics and efforts exerted by the Government of Sudan to address challenges of health worker migration within the framework of the WHO Code. Further, it derives lessons on WHO Code implementation and effectiveness to inform current debate and future steps on migration management in Sudan and beyond.

## Methods

This study was informed mainly by secondary analysis in terms of available literature and documentation on the issue, and mostly by unpublished material from local sources. Five key informant interviews were conducted involving senior personnel in the ministries of health, higher education, and labour. The analysis also benefited from the experience of the authors with migration issues over the past decade.

## Results

### The country and its health system

Sudan is the largest east African country, being nearly eight times the size of the United Kingdom, with an estimated population of 33 million in 2014 [4]. The country witnessed a protracted political and security turmoil that ultimately led to its southern part (one-fourth of its original size) to secede and vote for its independence in 2011. The loss of a considerable share of its oil resources following separation of the South exacerbated the economic difficulties in Sudan, with implications in several sectors, including health and social care. Out-of-pocket expenditure in the country is among the highest in the Eastern Mediterranean Region, ranging from 70 to 80 % [5].

The country did not achieve any of the Millennium Development Goals, the life span of which closes in 2015, although some progress with regards to reducing maternal mortality was achieved [4]. Communicable diseases still represent the major health burden in the country, although the incidence of non-communicable diseases is rising. Healthcare coverage is another major challenge, where disparities are well known and an estimated 14 % of the population lives with no access to health services [6]. The decentralized health system of the country is plagued by challenges related to governance, funding, health workforce, health information, service delivery, and access to drugs and technology.

Despite its huge educational potential represented by over 180 institutions including 35 medical schools, Sudan was classified in 2006 as a country with a critical shortage of health workers based on the WHO benchmark of 23 health workers per 10,000 population [7]. Other health workforce challenges include skill mix imbalances, inequitable geographic distribution and poor retention with intensive emigration. Health workforce governance, information systems and coordination were not strong enough to allow for robust planning and implementation of the required interventions [8].

A health system strengthening momentum initiated in 2001 brought more attention to health workforce issues. Capitalizing on this, the country implemented a number of important initiatives including upgrading the health workforce department of the Ministry of Health, establishment of the National Human Resources for Health Observatory and revitalization of health profession education including continuing professional development [9]. This has enabled some major achievements, including scaling up of health workforce production, with the number of nursing schools jumping from 18 in 2005 to 55 in 2012, expanding coverage of in-service training from 24 to 67 % of the health workforce over the same period, and improving geographic coverage through sanctioning of over 10,000 posts for states and rural areas [10]. These achievements, however, fall short of stabilizing the national health workforce due to remaining challenges including the high emigration rates.

### Sudan and health worker migration dynamics

**Fig. 1:**
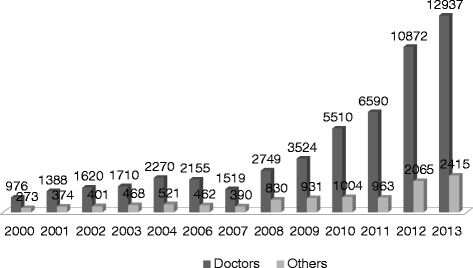
Records of experience certificates issued for health workers, 2000–2013. The records of the Documentation and Experience Certificate Office at the Federal Ministry of Health show a jump from 1249 to 15,352 certificates from 2000 to 2013

Health worker migration is a priority challenge in the country given that over half of its pool of medical doctors (composed of over 25,000) practices abroad and migration trends have been on the rise [11]. The past two decades witnessed an unprecedented out-flux of Sudanese health workers towards regional and international labour markets. Records of experience certificates issued for health workers by the Federal Ministry of Health (a strong proxy indicator for migration as these certificates are required to verify experience for purposes of working abroad) showed a jump from 1249 in the year 2000 to 15,352 certificates in 2013 (Fig. 1). The higher education sector is not exempt from the brain drain; it is estimated that Sudanese universities lost approximately 26 % of their teaching staff, with emigration rates reaching 40 % among the staff of the University of Khartoum, the prime higher education institution in the country [12].

Emigration of Sudanese health workers has traditionally been physician-led and male dominated. However, emerging trends reveal changes in gender migration with more participation of female doctors and nurses. Analysis of a cohort of 4200 Sudanese physicians recruited by the Ministry of Health of Saudi Arabia between 2010 and 2013 showed a female participation of 49 % [13], which corresponds to the trends observed in sub-Saharan African countries [14].

Traditional destinations for Sudanese migrants include the United Kingdom, the Republic of Ireland, and Libya, but recent out-flux has leaned towards the Gulf States, especially Saudi Arabia. Low remuneration, in addition to a lack of adequate opportunities for specialized training and career progression, are prominent push factors for migration [13]. Other associated push factors include issues related to work environment, lack of employment, poor management and security aspects. Better remuneration in the Gulf States is one major pull factor as the salary gradient for physicians reaches over 20 times that of Sudan [15]. Active recruitment through recruitment agencies has fuelled migration of Sudanese health workers, in particular to Saudi Arabia, over the past decade, leading to the recruitment of thousands of physicians and university academic staff [12].

The loss of huge numbers of health workers with essential expertise left a void and jeopardised both the coverage and quality of healthcare. Rural hospitals and primary healthcare centres suffered the most, but the adverse effects also inflicted some critical tertiary care areas. The loss of academic staff and trainers has resulted in serious gaps, with some training programs closing down or reducing their intake, especially for nursing, midwifery and allied health professions [12]. Based on widely felt implications, the migration of health workers became headline news in the media and a topical issue for debate in professional and public forums. Despite a vivid Sudanese diaspora and some positive returns in terms of technical capacity and remittances [11], the outlook of migration for Sudan is bleak. The continuing shed of key healthcare providers, health managers and the public health workforce currently represents the main challenge threatening the national health system.

### Migration evidence caveats and the role of the Observatory

As part of an overall weak health information system in the country, migration data and information suffer from scarcity, inaccuracy and fragmentation. There is no specific migration registry and studies on the phenomenon are few. With the rising public concern in relation to the observed out-flux of skilled labour from Sudan during the past decade, different estimates of the numbers of migrant health workers started to appear in the media and public forums. These estimates, ranging from very low to worrying figures [13], generated controversy and resulted in a ‘number debate’, creating some confusion and diverting attention from addressing the problem.

With the advent of the WHO Code, the already existing National Human Resources for Health Observatory (a multi-stakeholder network launched to generate health workforce data and coordinate partners) scaled up its efforts addressing the migration challenge. As the designated national authority for reporting on migration data, the National Human Resources for Health Observatory devoted a focus on migration evidence as part of its custodian role of the human resource information system. Thanks to this initiative, Sudan was among eight countries in the Eastern Mediterranean Region with designated national authorities and one of the only three countries that reported on migration to WHO [16]. The Observatory also spearheaded health workforce research and facilitated a number of studies on health worker migration. The emerging evidence fed into stakeholder meetings, public forums and the media, leading to professional and public momentum on the issue.

### Government approach to migration

Different governmental circles engaged in discussions regarding skilled worker migration and the Cabinet eventually constituted, in 2012, a high level committee to address the subject. Despite the lack of a solid national framework on the issue thus far, some remedial actions started to emerge in response to the challenges of migration. Media expressions from senior politicians, attention to migration evidence, and discussions around a policy on skilled worker migration are testimony of government attention to the issue. On the practical side, the national government recently doubled the remuneration package for university academic staff, expanded training capacity, and opted for signing bilateral agreements with some common destination countries for Sudanese migrants.

The government response, however, still falls short of the challenge with some worrying signs. Capitalizing on the lack of consistent evidence, some related governmental circles tend to underestimate the outflow of health workers. Other voices are overemphasizing the positive aspects of migration, viewing it as a complete gain for the country. Finally, a third faction demonstrates despair noting the inability of the country to bridge the salary gradient or counteract massive emigration. A senior government official stated, “*the salary gradient between us and countries in the Gulf is such huge that no effort at improving remuneration would affect migration decisions*”. A state minister maintained, “*there should be no worry about migration, we will produce more to compensate for the loss*”. The overall result is an unjustified lack of a national policy on health worker migration in the country in the midst of this huge unregulated migratory flow.

### The labour market and recruitment agencies

The health labour market in Sudan is complex and includes a number of challenges. Misalignment of health worker education and employment capacity with the healthcare needs of the country is problematic, resulting in underemployment and unemployment even among vital categories such as physicians and nurses [17]. These imbalances are no doubt a trigger for out-migration and the situation is further exacerbated by the advent of recruitment agencies that facilitate migration and even mobilize experienced, highly skilled workers through active recruitment for positions in the affluent Gulf States [11]. According to Ministry of Labour statistics, the number of recruitment agencies increased from less than 10 in the year 2000 to approximately 400 in 2013. Despite their role in facilitating the professional integration of Sudanese migrant health workers abroad, the practice of these recruitment agencies has generated ethical dilemmas and it is not uncommon to see media coverage of stories around illegal and fraudulent conduct affecting those intending to migrate.

### Destination country response

The WHO Code provides a framework for source and destination countries to enter into bilateral agreements to manage migration for mutual gain while observing the rights of health workers. The experience of Sudan demonstrates the challenging side of this story. The government of Sudan exerted efforts to sign bilateral agreements with Saudi Arabia and Libya, two main destination countries. Negotiations continued for over 3 years and the agreements were finally signed. However, the agreements were not effectively implemented and migratory flows from Sudan to the two countries, as well as other destinations, remained largely unmanaged. No form of remuneration, whether financial or technical, was accrued by Sudan for losing its substantial investment in its flying human capital.

## Discussion

The Government of Sudan’s response to health worker migration has evolved over the past decade from one of neglect to one of attention and subsequent active involvement. As a long-standing phenomenon, migration of health workers and skilled personnel in general has not been a topic of focus for the government. However, the rising professional and media concern over the past few years, in addition to the advent of the WHO Code, brought the issue to centre stage. The advocacy role played by the Observatory and other entities, including the media, has been instrumental in sensitizing the government and providing a framework for migration management in line with the WHO Code stipulations.

The WHO Code uptake by the Government of Sudan has, nevertheless, been beneficial to some degree. In addition to fostering some key interventions, such as the signing of bilateral agreements with source countries, the WHO Code also helped boost the broader health workforce development efforts in Sudan. The development of the first-ever national health workforce strategy in the country in 2012 was informed by the WHO Code, which has proven useful in strengthening the human resource information system. In 2011, Sudan received a grant from the Global Fund to Fight AIDS, Tuberculosis and Malaria supporting health workforce research and studies related to migration guided by the relevant provisions of WHO Code.

However, there have also been limitations in the uptake of the WHO Code in Sudan. Despite advocacy efforts, the publicity of the WHO Code was not effective enough to influence key aspects such as legislation and ethical recruitment practices. Actions taken by some stakeholders, including civil service, recruiters and private sector, were not satisfactory. Comparatively, other countries, such as the Philippines, managed to achieve better results in WHO Code implementation through effective stakeholder involvement, orientation and capacity building, and compliance with ethical recruitment guidelines [18].

The outlook for effective migration management through bilateral agreements in Sudan is bleak. A senior Sudanese official reflected on the lack of implementation of bilateral agreements by stating that, “*as far as the WHO Code is voluntary and as far as beneficiary countries in the region do not have media or civil groups pressures, they will not be part of this code, I do not think there is hope! *”. Several factors are arguably behind this lack of bilateral agreement implementation. Apart from the ‘ethical call’, the negotiation power of Sudan as a source country has always been weak due to the rampant push factors that render health workers prepared to accept international employment. This is opposed to a privileged position of destination countries that are in need of those skills and possess the economic and professional attraction to pull qualified health workers. Additionally, destination countries, especially the Gulf States, experience a ‘free rider’ effect by recruiting expatriates through direct individual contracts without concerning themselves over binding agreements with source countries. Unlike other destination countries such as Norway and Switzerland [18], the Gulf States have not implemented effective steps towards self-sufficiency and development of the domestic health workforce and hence continue to rely heavily on expatriate health workers.

Political sensitivities are generally tied to dialogue around migration in the Middle East and Arab region, especially in terms of discussing issues relating to managing flows and addressing equal employment and conditions of work for expatriates. This is reflected in the language and outcomes of several attempts to address the issue, including a recent conference in the region on health worker migration which called on implementing the WHO Code [19].

Generally speaking, and particularly in the case of Sudan, the role of relevant regional and international agencies in catalysing migration management is lacking. For instance, there was no regional framework to support Sudan’s efforts in signing bilateral agreements with destination countries and a mediation role was absent. There was also no strong advocacy role to emphasise the ethical aspect and solidarity in relation to implementing the WHO Code. Nevertheless, some international agencies and development partners have supported steps to strengthen the health workforce and mitigate adverse effects of migration in Sudan and other countries. The approach adopted by the Global Fund to Fight AIDS, Tuberculosis and Malaria and the Global Alliance for Vaccines and Immunization to move from funding disease-specific interventions to supporting broader health system and health workforce strengthening is a best practice example in this front.

## Conclusions

Sudan has taken measures to address the challenges of health worker migration and the advent of the WHO Code has catalysed some vital steps in managing migration and strengthening the national health workforce. Yet, the country’s response falls short of the effective measures required to address migration and to utilize the WHO Code to its full potential. The experience of Sudan reflects the complexity of health workforce mobility and the issues involved within and beyond the country context with several emerging lessons. Improving the evidence base, harnessing the national dialogue to implement appropriate national policies, and strengthening regulatory frameworks in addition to streamlining stakeholders are vital pre-requisites for an effective country response to migration. Another main lesson is that migration management is never a country-to-country issue; there must be advocacy and catalytic roles for relevant regional and international agencies if efforts are to succeed using less binding tools such as the WHO Code.

Revisions of the WHO Code should consider improving advocacy, promoting a health diplomacy approach, introducing more measures to strengthen the global health workforce, and allowing a more prominent role for relevant regional and international entities. The WHO Code is relevant but its effectiveness would benefit highly from the lessons learned following its implementation over the past few years.
